# Purification of the Mails

**Published:** 1904-07

**Authors:** 


### Purification of the Mails.

THE government of America is not oppressive. Complaint is seldom made that our people are governed too much. Our laws are human and beneficent; this is not surprising, as we make them ourselves. This is true of our laws in general and it is also true with greater particularity of the law for the protection of the health of the people known as the public health law.

Among others of its important provisions the public health law prescribes who may and who may not practise medicine in this state, and as the practice of medicine has ever been a reasonably lucrative vocation, its opportunities for money making have always been attractive. Again, the practice of medicine offers limitless opportunities for the exhibition of the devices of the fakir. All men and some women are credulous,—none more so than the sick, especially those incurably or hopelessly afflicted.

For many centuries a vast and varied horde of grafters, looters, fakirs, boy wonders, camp followers and healers have preyed upon and plundered the credulous and the sick with promises of healing,—healing swift and sure. The arts and the devices of this class of criminals depend largely upon advertising,—advertising in every possible way their audacious and mendacious claims. Cheap wood pulp paper and the omniverous all-devouring rotary printing press, have made the fake healer possible and often profitable.

For centuries the profession as individuals and as membership corporations has protested, has warned, has admonished. At length it would seem that a brighter and better day is at hand. The American government has given notice that its mails cannot be longer used to foster and promote the fraudulent operations of the medical fakirs and imposters; that colored water cures for consumption and cancer are frauds and their advertisements unmailable; that sure cures for the opium and whiskey habits, which contain large quantities of morphia and alcohol, are frauds and the parties to its propagation are criminals, and the newspapers printing their advertisements are unmailable; that invitations to unfortunate girls and still more foolish or wicked wives to have abortions procured "while you wait" are contrary to public policy, and that the abortion trade can no longer use the mails for correspondence or for distributing papers containing such advertisements.

We are confident no more important step in the interest of the public health, of general morality and of the well-being of the American people has been taken or will be taken during the century. The administration is wise and will prosper exceedingly which dares and does so much. The American Medical Association at its recent meeting took action commendatory of this important step which is being taken by the postoffice department at Washington.

